# Treatment of Contaminated Groundwater via Arsenate Removal Using Chitosan-Coated Bentonite

**DOI:** 10.3390/molecules24132464

**Published:** 2019-07-04

**Authors:** Jurng-Jae Yee, Carlo Vic Justo Arida, Cybelle Morales Futalan, Mark Daniel Garrido de Luna, Meng-Wei Wan

**Affiliations:** 1 Department of Architectural Engineering, Dong-A University, Saha-gu, Busan 49315, Korea; 2 Environmental Management Bureau, Department of Environment and Natural Resources, Quezon City 1100, Philippines; 3 National Research Center for Disaster-Free and Safe Ocean City, Dong-A University, Busan 49315, Korea; 4 Department of Chemical Engineering, University of the Philippines Diliman, Quezon City 1101, Philippines; 5 Department of Environmental Resources Management, Chia Nan University of Pharmacy and Science, Tainan 71710, Taiwan

**Keywords:** activation energy, arsenic, bentonite, chitosan, groundwater, thermodynamics

## Abstract

In the present research, treatment of contaminated groundwater via adsorption of As(V) with an initial concentration of 50.99 µg/L using chitosan-coated bentonite (CCB) was investigated. The effect of adsorbent mass (0.001 to 2.0 g), temperature (298 to 328 K), and contact time (1 to 180 min) on the removal efficiency was examined. Adsorption data was evaluated using isotherm models such as Langmuir, Freundlich, and Dubinin-Radushkevich. Isotherm study showed that the Langmuir (*R*^2^ > 0.9899; *χ*^2^ ≤ 0.91; *RMSE* ≤ 4.87) model best correlates with the experimental data. Kinetics studies revealed that pseudo-second order equation adequately describes the experimental data (*R*^2^ ≥ 0.9951; *χ*^2^ ≤ 0.8.33; *RMSE* ≤ 4.31) where equilibrium was attained after 60 min. Thermodynamics study shows that the As(V) adsorption is non-spontaneous (Δ*G*^0^ ≥ 0) and endothermic (Δ*H*^0^ = 8.31 J/mol) that would result in an increase in randomness (Δ*S*^0^ = 29.10 kJ/mol•K) within the CCB-solution interface. FT-IR analysis reveals that hydroxyl and amino groups are involved in the adsorption of As(V) from groundwater. Results of the present research serve as a tool to determine whether CCB is an environmentally safe and cost effective material that could be utilized in a permeable reactive barrier system for the remediation of As(V) from contaminated groundwater.

## 1. Introduction

Arsenic contamination in water bodies has become a serious environmental problem worldwide. Arsenic is a metalloid characterized by its carcinogenic properties and acute toxicity [1]. More importantly, arsenic has been classified as a Class A carcinogen according to the International Agency for Research on Cancer [2]. The occurrence of arsenic in groundwater and surface water resources could be attributed to natural processes including weathering of arsenic sulfide minerals and dissolution of arsenic-containing oxyhydroxides and iron oxides under reducing conditions [3,4]. Anthropogenic activities such as agriculture, mining, and burning of fossil fuels, production of herbicides and pesticides, semiconductor industries, aquaculture, hydraulic fracturing, and smelting of non-ferrous metals are considered major sources of arsenic [5,6,7].

In the past decades, arsenic contamination has been widely reported in various countries, including India, China, Bangladesh, Vietnam, Cambodia, New Zealand, Philippines, United States, and Taiwan [8,9,10,11]. The consumption of arsenic-contaminated water on a long-term basis has been known to cause cell proliferation, oxidative stress, and methylation of altered DNA that could lead to cancer of the kidney, liver, prostate, rectum, skin, and urinary tract [12,13]. Other diseases related to the prolonged exposure to arsenic are diabetes mellitus, hypertension, dermal lesions, hyperkeratosis, peripheral neuropathy, loss of appetite, gangrene of the limbs, and dysfunction of the cardiovascular, cerebrovascular, and respiratory system [14,15,16]. In addition, exposure of aquatic organisms to low concentration of arsenic and selenium has been known to cause various types of cancers and mutagenesis [17]. Stringent regulations have been adopted by the United States Environmental Protection Agency, World Health Organization, Taiwan, and the European Commission that set the maximum standard concentration of arsenic at 10 μg/L in drinking water [18,19,20].

Groundwater systems typically contain inorganic arsenic species in the form of arsenite [As(III)] with an oxidation state of +3 and arsenate [As(V)] with a oxidation state of +5 [21]. Typically, As(V) exists as H_2_AsO_4_^−^, HAsO_4_^2−^, and AsO_4_^3−^ in groundwater under oxidizing conditions within pH range of 2 to 12. Meanwhile, As(III) as H_3_AsO_3_ predominates under reducing, anaerobic conditions within a pH range of 2 to 9 [22].

Technologies including conventional pump-and-treat method and permeable reactive barrier (PRB) system are applied in the treatment of contaminated groundwater. There are several disadvantages associated with the pump-and-treat method including high capital cost for the installation and maintenance as well as inefficiency in removing contaminants [23]. Recently, PRB system has gained popularity due to its ability to treat a wide range of pollutants, reduced operational costs, and in-situ immobilization of contaminants [24]. A PRB is a passive treatment system where a contaminated plume moves via natural gradient through the material barrier [25]. The selection of a reactive material to be utilized in a PRB system is one of the major design considerations. Activated carbon, zeolites, hydrated lime, and zero valent iron are commonly used as reactive materials in a PRB [25,26].

The removal of pollutants using PRB involves a combination of adsorption, biodegradation, and precipitation. Adsorption onto solid materials is considered the main removal mechanism of arsenic-contaminated plumes. It is an attractive technology due to its cost-effectiveness, easy operation, and low maintenance. Moreover, it is applicable in household module and industrial plants and has the capacity for adsorbent regeneration [27,28]. Extensive literature review shows several materials have been utilized in arsenic and radioactive elements removal including magnetic metal-organic framework nanocomposite [29], crosslinked chitosan/MMT [30], chitosan-coated bentonite [31], clinoptilolite-rich tuff [32], activated bauxite [33], hematite [33], kaolinite [34], montmorillonite [34], granular ferric oxide [35], UltraCarb [36], nZVI-zeolite [37], F400 [38], polymer-clay nanocomposite ion exchange resin [39], and iron-titanium oxide [40]. Spent adsorbents often undergo a regeneration process or may require special handling before disposal. Previous studies have utilized spent adsorbents as lightweight aggregates or thermal insulators in cement mortars [41,42].

Recently, chitosan-clay composites have gained attention due to its biodegradability, low-cost, eco-friendliness, excellent adsorption capacity, improved heat resistance, and enhanced mechanical stability [43,44]. Natural, solid waste products as adsorbents have been regarded as an attractive alternative to commercial adsorbents. Chitosan is a renewable resource where it can be derived from the by-product of seafood manufacturing specifically the exoskeletons of crustaceans [45,46]. Meanwhile, clay materials are produced by mining activities as overburden wastes [47]. Chitosan is a cationic, heterogeneous polymer characterized by its non-toxicity, biocompatibility, hydrophilicity, and abundance of amino and hydroxyl groups [48,49,50]. The applicability of clay materials in the remediation of heavy metals is attributed to its lamellar structure, antimicrobial activity, high cation exchange capacity, abundance, low-cost, and high surface area. Most importantly, disposal of clay materials does not have any detrimental effect on the environment [51,52]. Several reports have investigated the removal of arsenic from aqueous solution using different chitosan-clay composites including crosslinked chitosan/montmorillonite [30], chitosan/clay/magnetite composite [53], and chitosan-coated bentonite (CCB) [31,54]. Arida et al. (2016) [31] reported the maximum breakthrough capacity of 10.57 μg/g for CCB in the removal of As(V) under fixed-bed conditions. Batch studies have been performed by Futalan et al. (2019) [54] to determine the adsorption capacity of CCB in arsenic removal from aqueous solution. However, previous researches have investigated arsenic removal using CCB in synthetic solutions. There are no published studies on the utilization of CCB in the removal of As(V) from contaminated groundwater. It is essential for studies to deal with real wastewater or groundwater that could provide relevant data for treatment technologies.

As part of the extended research of Arida et al. (2016) [31] and Futalan et al. (2019) [54], the present study utilizes static experiments to examine the removal and mechanism of As(V) from contaminated groundwater using CCB. Equilibrium data was evaluated using isotherm models including Dubinin-Radushkevich, Freundlich, and Langmuir. The thermodynamic parameters including activation energy, entropy, enthalpy, and Gibbs free energy were evaluated while adsorption kinetics was investigated using intraparticle diffusion, pseudo-second order, film diffusion, and pseudo-first order equations. Characterization of CCB using scanning electron microscopy (SEM) and Fourier transform infrared spectroscopy (FT-IR) was performed.

## 2. Materials and Methods

### 2.1. Materials

Bentonite (Al_2_H_2_Na_2_O_13_Si_4_), low-molecular weight chitosan (75–85% deacetylation; MW = 50 to 190 kDa), sodium hydroxide (NaOH, ≥95%), hydrochloric acid (HCl, 37% fuming), and nitric acid (HNO_3_, 65% *w*/*w*) were procured from Merck (Darmstadt, Germany). All reagents utilized in the study were of analytical grade. Arsenic contaminated groundwater was collected from a monitoring well located in Tainan, Taiwan. The physico-chemical characteristics of the groundwater including As(V) concentration are presented in Table 1.

### 2.2. Characterization

The surface morphologies of bentonite, chitosan, and CCB were obtained using SEM (S-3000N Hitachi, Tokyo, Japan) under a vacuum atmosphere of 1.33 × 10^−6^ mBar and voltage of 20 kV. Samples of CCB before and after adsorption were analyzed using FT-IR (Jasco FT-IR 410, Tokyo, Japan) with 64 scans and 4 cm^−1^ resolution within wavelength of 4000–400 cm^−1^.

### 2.3. Synthesis of CCB

The method utilized was adapted from the procedure performed by Wan et al. (2010) [55]. About 5 g chitosan was mixed with 100 mL 5% (*v*/*v*) HCl, and the mixture was stirred for 2 h at 300 rpm. Then, 100 g bentonite was added into the solution and stirred for 3 h. Adjustment of pH was performed by adding 1 N NaOH drop by drop. The CCB particles were allowed to settle via sedimentation where the beads were repeatedly washed using deionized water until neutral pH was attained. Finally, the beads were dried at 65 °C using an oven (Channel Precision DV452 200 V, Taipei, Taiwan) for 24 h.

### 2.4. Adsorption Experiments

Kinetic studies were performed where 1.5 g CCB and 30 mL groundwater in a 125-mL Erlenmeyer flask were agitated using a reciprocal shaker bath (BT-350, Tainan, Taiwan) at 25 °C and 50 rpm. Under pre-determined time intervals (1 min to 3 h), treated samples were collected and filtered (Whatman 40, Merck, Darmstadt, Germany). The filtrate was analyzed for residual As(V) concentration using inductively-coupled plasma optical emission spectrophotometer (ICP-OES, Perkin-Elmer DV2000, Wellesley, MA, USA).

Varying mass of CCB (0.001 to 2.0 g) was utilized to carry out equilibrium experiments. In the study, groundwater (30 mL) with 50.99 μg/L As(V) was agitated at 50 rpm and 25 °C for 1 h.

Thermodynamic studies were performed using 1.5 g CCB and 30 mL groundwater with an initial As(V) concentration of 50.99 μg/L. The solution was agitated at 50 rpm for 1 h while the temperature was varied from 298 to 328 K.

### 2.5. Error Analysis

The suitability of the models with the experimental data was evaluated using error analysis. Root mean square error (*RMSE*) and chi-square (*χ*^2^) are represented as Equations (1) and (2):

(1) $$RMSE=\sqrt{\left(\frac{1}{N}\sum_{i=1}^{N}{\left(q_{theo,i}-q_{\exp,i}\right)}^{2}\right)},$$

(2) $$\chi^{2}=\sum \left(\frac{{\left(q_{\exp}-q_{theo}\right)}^{2}}{q_{theo}}\right)$$

where *q_theo_* refers to the adsorption capacity generated by the model (mg/g), *q_exp_* refers to the experimental adsorption capacity (mg/g), *N* is the total number of experimental data [56,57]. A *RMSE* and *χ*^2^ value close to zero suggest that the model utilized is a better fit and more reliable in the prediction of theoretical values wherein a lower bias exists between the predicted and experimental values [58,59].

## 3. Results and Discussion

### 3.1. Characterization

**Figure 1:**
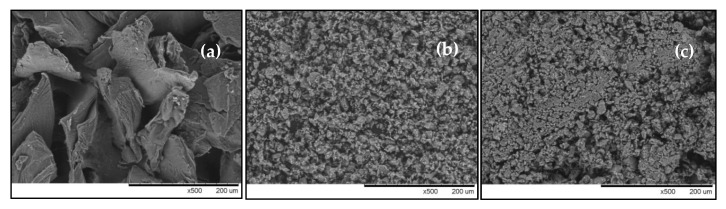

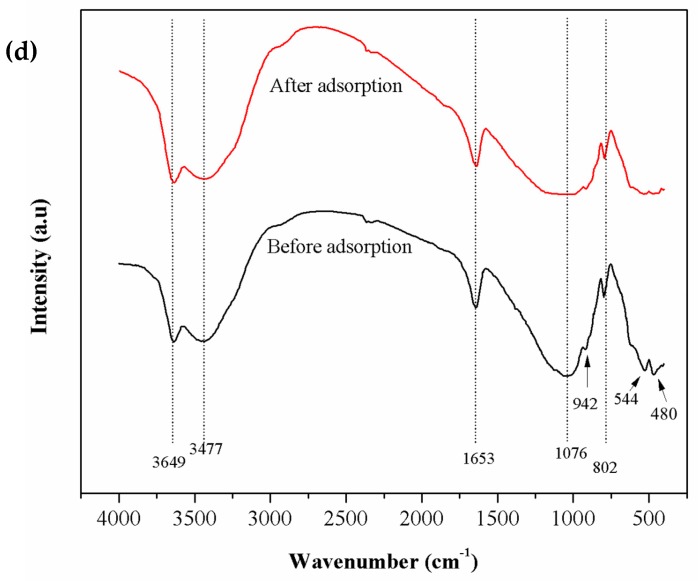
SEM micrographs of (**a**) chitosan, (**b**) bentonite, and (**c**) chitosan-coated bentonite (CCB) at 500× magnification, and (**d**) FT-IR spectra of CCB before and after adsorption.

Figure 1 shows the surface morphology of chitosan, bentonite, and CCB. Chitosan exhibits a smooth, homogenous, solid mass with flaky appearance (Figure 1a). The morphology of bentonite is comprised of smaller particles with a rough and porous structure (Figure 1b). Figure 1c shows the surface of CCB to be less porous in comparison to bentonite.

FT-IR spectra of CCB before and after adsorption are displayed in Figure 1d. Several peaks are attributed to bentonite: 3649 cm^−1^ refers to the Si–OH and Al–OH group stretching vibration, 668 cm^−1^ indicates Si–O bond bending, 544 cm^−1^ for bending vibrations of the Si–O–Si and metal oxide bond (Al–O–Si) is represented by 480 cm^−1^ for bending vibrations [60,61]. In chitosan, the characteristic bands are the following: 3477 cm^−1^ refers to the –NH and –OH stretching vibrations, 2914 cm^−1^ refers to aliphatic C–H stretching vibrations, 1653 cm^−1^ suggests amide I–NH bending vibration in –NH_2_, 1373 cm^−1^ refers to deformation vibration of –NH in –NH_2_ and 1076 cm^−1^ is attributed to stretching vibration of –CO in –COH [62]. After adsorption of As(V), the following peaks appeared to have shifted from 3649 to 3642 cm^−1^ (–OH stretching vibrations) and 3477 to 3471 cm^−1^ (–OH and –NH_2_ stretching). This indicates the involvement of –OH groups of CCB in the uptake of As(V) from groundwater.

### 3.2. Effect of Adsorbent Mass, Contact Time, and Temperature

**Figure 2:**
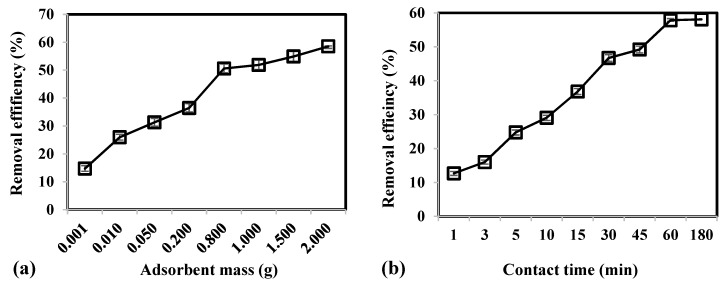

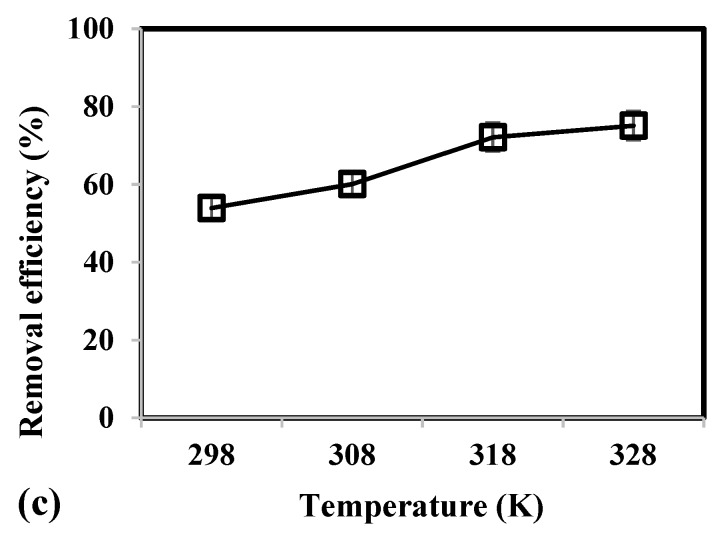
Effect of (**a**) adsorbent mass, (**b**) contact time, and (**c**) temperature on the removal efficiency of As(V) from groundwater using CCB.

Figure 2 illustrates the parametric effects of adsorbent mass, contact time, and temperature on the removal efficiency of As(V) using CCB. Results demonstrate that increasing adsorbent mass from 0.001 to 2.0 g led to an increase in removal efficiency from 14.70% to 58.55%, respectively. A higher amount of adsorbent present in the system would imply that there is greater surface area and higher number of binding sites that are available for adsorption.

Adsorption of As(V) was rapid within the first 30 min, which attained a removal of 46.70% (Figure 2b). Initially, high concentration gradient and availability of numerous binding sites on the CCB surface cause the fast uptake of As(V) [63]. As adsorption proceeds, a gradual increase in removal from 46.70% to 58.09% was observed as contact time was increased further from 30 to 180 min. This could be attributed to the saturation of binding sites on the CBB surface that would require As(V) ions to diffuse into the pores of CCB. Moreover, As(V) ions adsorbed onto CCB surface exert repulsive forces to the approaching As(V) in the bulk phase [64]. It was observed that equilibrium was attained at 60 min with a removal of 57.84%.

In Figure 2c, results show that increase in temperature from 298 to 328 K caused a corresponding increase in removal efficiency from 53.92% to 75.09%, respectively. The uptake of As(V) was favored at higher temperatures due to the increase in kinetic energy that would allow ions to quickly diffuse from bulk of solution to the surface and pores of CCB. Moreover, a higher temperature of 328 K would lead to lower viscosity of the solution and greater rate of collision between the binding sites of CCB and As(V) [63,65].

### 3.3. Kinetic Study

The kinetic data was fitted using the intraparticle diffusion, pseudo-second order, pseudo-first order, and film diffusion equation. The linear forms of the intraparticle diffusion [66], pseudo-second order equation [67], Lagergren or pseudo-first order equation [68], and film diffusion equation [69] are provided as Equations (3)–(6):

(3) $$q_{t}=k_{\mathrm{int}}t^{0.5}+C,$$

(4) $$\frac{t}{q_{t}}=\frac{1}{k_{2}q_{e}{}^{2}}+\frac{t}{q_{e}},$$

(5) $$\ln\left(q_{e}-q_{t}\right)=\ln q_{e}-\frac{k_{1}}{2.303}t,$$

(6) $$\ln(1-\frac{q_{t}}{q_{e}})=-k_{fd}t,$$

where ***k*_1_** is the pseudo-first order rate constant (min^−1^), ***k*_2_** is the pseudo-second order rate constant (g/mg•min), ***k_int_*** is the intraparticle diffusion rate constant (mg/g•min^0.5^), ***k_fd_*** refers to the rate constant in film diffusion (min^−1^), ***C*** refers to the boundary layer thickness, ***q_t_*** and ***q_e_*** is the adsorption capacity at time ***t***, and equilibrium (mg/g), respectively.

**Figure 3:**
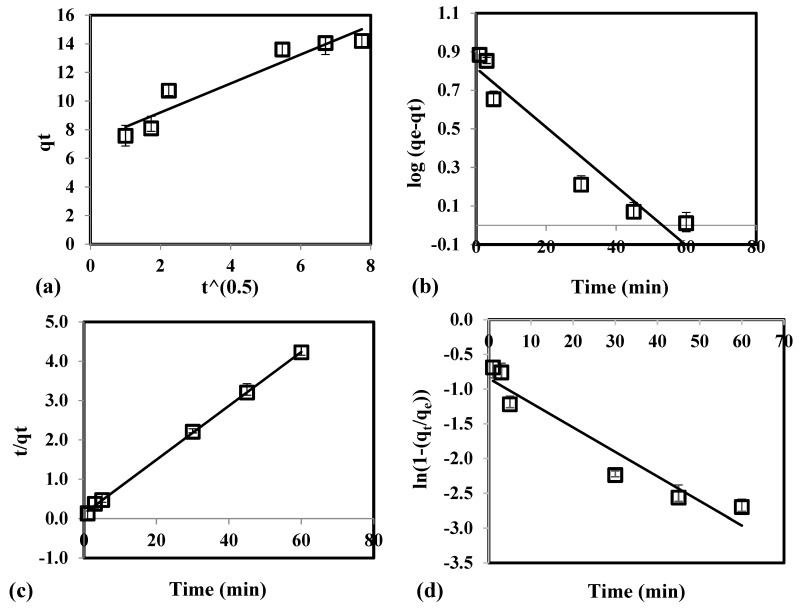
Experimental data fitted with (**a**) intraparticle diffusion, (**b**) pseudo-first order, (**c**) pseudo-second order, and (**d**) film diffusion equation for the adsorption of As(V) using CCB.

In Figure 3a, the linear plot of intraparticle diffusion equation did not pass through the origin. This signifies that intraparticle diffusion is not the rate-determining step of the adsorption system. In Table 2, the high value of coefficient of determination (*R*^2^ ≥ 0.9951) and low values for *RMSE* (≤ 4.31) and *χ*^2^ (≤ 8.33) for pseudo-second order equation were obtained. Experimental *q_e_* (13.61 mg/g) was observed to have similar value with the theoretical *q_e_* generated by the pseudo-second order model. In addition, the linear plot generated by the pseudo-second order (Figure 3c) fitted better with the experimental data than intraparticle diffusion (Figure 3a), pseudo-first order (Figure 3b), and film diffusion (Figure 3d). This implies that pseudo-second order equation adequately describes the As(V) adsorption from groundwater using CCB. Moreover, it indicates that the adsorption system has chemisorption as its rate-limiting step where sharing or exchange of electrons occurs between the contaminant and CCB.

### 3.4. Isotherm Study

Isotherm studies determine the interaction between the adsorbate and adsorbent. The adsorption characteristics at equilibrium were assessed with Freundlich [70], Langmuir [71], and Dubinin-Radushkevich (D-R) [72] models, where the linear forms are presented as Equations (7)–(9):

(7) $$\log q_{e}=\log K_{F}+\frac{1}{n}\log C_{e},$$

(8) $$\frac{1}{q_{e}}=\frac{1}{K_{L}q_{m}C_{e}}+\frac{1}{q_{m}},$$

(9) $$\ln q_{e}=\ln q_{DR}-\beta \varepsilon^{2},$$

where *q_m_* (mg/g) is the maximum adsorption capacity at monolayer coverage, *K_L_* (L/mg) is the Langmuir energy constant attributed to heat of adsorption, *n* (g/L) refers to the linearity deviation of adsorption and heterogeneity factor, *K_F_* (mg/g) refers to the Freundlich adsorption capacity, *q_DR_* (mg/g) is the monolayer capacity derived from D-R isotherm, *ε* refers to the Polyani potential, and *β* (mol^2^/kJ^2^) refers to the adsorption energy constant. The parameter *E* (mean adsorption energy, kJ/mol•K) and *ε* can be calculated using Equations (10) and (11):

(10) $$\varepsilon =RT\ln\left[1+\frac{1}{C_{e}}\right],$$

(11) $$E=\frac{1}{\sqrt{2\beta }},$$

where *T* (K) refers to the absolute operating temperature and *R* (8.314 kJ/mol•K) refers to the universal gas constant. The value of *E* defines the transfer of one free energy of solute from solution onto adsorbent surface [73].

**Figure 4:**
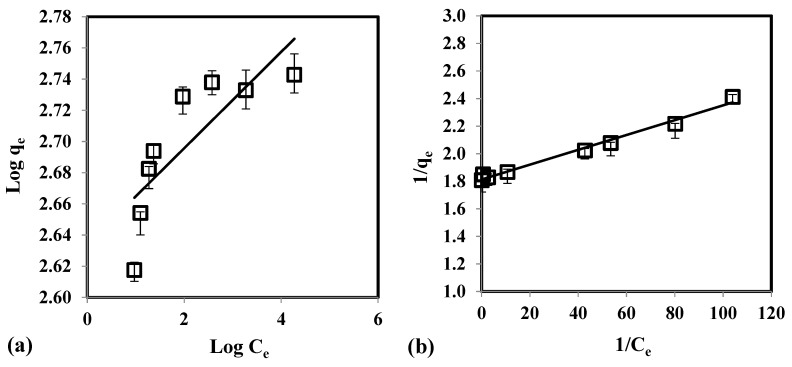

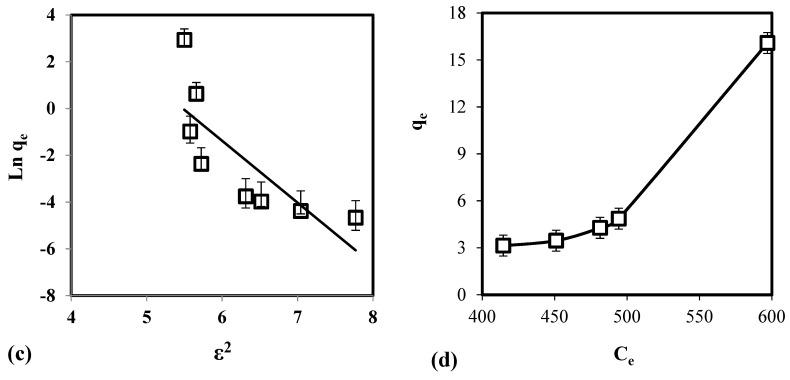
Equilibrium data fitted with (**a**) Freundlich, (**b**) Langmuir, (**c**) D-R, and (**d**) solvent-affinity isotherm for the adsorption of As(V) using CCB.

**Figure 5:**
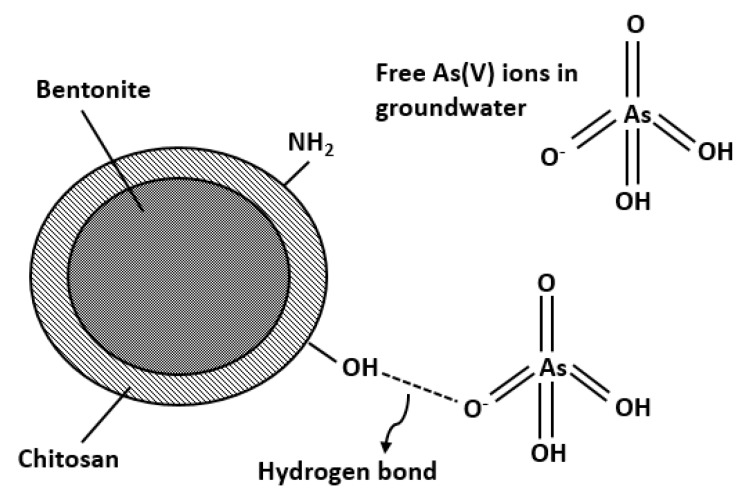
Adsorption of mechanism of As(V) onto CCB surface in groundwater.

Table 3 illustrates the calculated regression analysis parameters (*χ*^2^, *RMSE*, *R*^2^) and isotherm parameters. Based on the *R*^2^ and error analysis values, the uptake of As(V) follows the Langmuir model (*R*^2^ ≥ 0.9688; *RMSE* ≤ 4.87; *χ*^2^ ≤ 0.91). Moreover, the linear form of Langmuir isotherm is in good agreement with the equilibrium data (Figure 4). This indicates that the removal of As(V) occurs as monolayer adsorption onto binding sites with homogeneous energy levels. Figure 4d illustrates that the isotherm has a concaved/curved upward shape otherwise known as the solvent-affinity isotherm. This is further validated by the Freundlich constant *n*, which has a value of less than unity. This indicates that increase in surface concentration results in the increase of marginal sorption energy that indicates a strong intermolecular attraction within the adsorbent layers [74,75]. The *E* value (*E* = 0.9077 kJ/mole) derived from D-R isotherm implies that the removal of As(V) follows physical adsorption [76]. Adsorption of As(V) onto CCB is described in the following mechanism. Previous study of Calagui et al. (2014) determined the isoelectric point of CCB to occur at pH = 2.8 [77]. In groundwater, As(V) can be found in two dissociated complex forms (H_2_AsO_4_^−^ and HAsO_2_^2^) while CCB would have a negative surface charge due to its hydroxyl (–OH) groups taking the form of –O−. This would facilitate hydrogen bond formation between surface hydroxyl of CCB and –OH of As(V) molecule [78]. Adsorption may also occur due to the coordination between –OH ligands of the As(V) molecule and hydroxyl groups of CCB (Figure 5) [79].

The adsorption capacity obtained in the present research was compared with previous adsorbents (Table 4). In general, CCB has lower adsorption capacity to other adsorbents reported in literature with the exception of F400. Commercial adsorbents were observed to have greater adsorption capacity than CCB, which ranges from 38.3 to 55.0 mg/g. Adsorbents such as CCB and F400 were tested in real groundwater while other adsorbents were applied in aqueous or synthetic solution. Natural groundwater contains a complex matrix of anions and cations that could lead to the suppressed adsorptive performance of CCB in the uptake of As(V). Decreased adsorption capacity of CCB could be due to the inhibitive effect of existing anions (phosphate (PO_4_^3−^), chloride (Cl^−^), and sulfate (SO_4_^−^)) in groundwater that would compete for the available adsorption sites of CCB for the uptake of As(V) [80]. Previous studies have reported that the presence of PO_4_^3−^ at high concentration would hinder the removal of As(V) due to similar behavior towards the binding sites of the adsorbent. The molecular structure and aqueous chemistry of As(V) and PO_4_^3−^ are similar where both species form tetrahedral oxyanions [19,81].

In addition, the presence of cations such as K^+^, Ca^2+^, Na^+^, and Fe^2+^ can lead to retention of negatively-charged As(V) in the groundwater instead of being adsorbed onto the CCB surface. Groundwater also contains humic acid, which is a hydrophilic organic acid of dispersive molecular weight. The slightly basic pH of groundwater would imply negatively-charged humic acid due to its hydroxyl and carboxyl groups. The inhibited removal of As(V) could be attributed to the competition between humic acid and As(V) for the adsorption sites on the CCB surface [82].

### 3.5. Thermodynamics Study

Adsorptive removal of As(V) using CCB under varying temperature was assessed by determining the thermodynamics parameters. The free Gibbs energy (Δ*G*^0^) is given by Equations (12) and (13):

(12) $$\Delta G^{0}=-RT\ln K,$$

(13) $$K=\frac{C_{ads}}{C_{e}},$$

where *C_ads_* refers to the quantity of As(V) adsorbed by CCB (mg/L) and *K* refers to the equilibrium constant. The parameters entropy (Δ*S*^0^) and enthalpy (Δ*H*^0^) are derived using the Van’t Hoff equation (Equation (14)):

(14) $$\ln K=\frac{\Delta S^{0}}{R}-\frac{\Delta H^{0}}{RT}.$$

Activation energy (*E_a_*, kJ/mol) refers to the minimum energy that contaminant molecules have to overcome for adsorption to occur [59]. The Arrhenius equation is given as Equation (15):

(15) $$\ln k_{2}=\ln A-\frac{E_{a}}{RT}$$

where *k*_2_ is the pseudo-second order rate constant and *A* is the frequency factor or pre-exponential factor [83].

From Table 5, the positive *E_a_* value indicates that there is a need to overcome an energy barrier by increasing the temperature of the adsorption system. The magnitude of *E_a_* would define the form of adsorption governing the process, whether it is physisorption (5 ≤ *E_a_* < 20 kJ/mol) or chemisorption (≥40 kJ/mol) [84]. Based on the *E_a_* value of 14.72 kJ/mol the governing mechanism is physisorption.

The removal of As(V) is an endothermic process based on the positive value of Δ*H*^0^. This also suggests that the degree of total energy released during bond formation is less than the degree of total energy captured during bond breakage [83]. Depending on the value of Δ*H*^0^, the adsorption system could be described as physical (<21 kJ/mol) or chemical (~100–500 kJ/mol) in nature [85]. Based on the magnitude of Δ*H*^0^, removal of As(V) using CCB is governed by physisorption, which agrees with the obtained *E_a_* value. The positive Δ*S*^0^ value signifies occurrence of dissociative mechanism that would lead to an increase in randomness after As(V) was adsorbed from solution onto the CCB surface [59]. Moreover, a positive Δ*S*^0^ value would imply a good affinity between As(V) and binding sites of CCB [86]. Under all temperature, the positive values of Δ*G*^0^ indicate the non-spontaneity of the adsorption system using CCB. Adsorption is favored at higher temperature since Δ*G*^0^ values were observed to become less positive as temperature was increased from 298 to 328 K, implying that adsorption becomes more non-spontaneous at lower temperature.

## 4. Conclusions

Under static conditions, the present study evaluated the adsorptive capacity of CCB for the treatment of As(V) in contaminated groundwater. Equilibrium data followed the Langmuir model with maximum adsorption capacity of 1.47 mg/g at monolayer coverage, which implies CCB uptake of As(V) occurs onto active sites with homogeneous energy levels. Kinetic studies determined that the adsorption system can be best described using pseudo-second order equation that indicates that the rate-limiting step is chemisorption. Based on the thermodynamics data, the adsorption system is endothermic, non-spontaneous and results to an increased randomness of As(V) as it is adsorbed from solution onto CCB surface. FT-IR analysis shows that uptake of As(V) involves binding sites such as hydroxyl (–OH) and amino (–NH_2_) groups. In general, the removal of As(V) using CCB from groundwater is a combination of physical and chemical adsorption, where its main mechanism is physisorption based on the *E_a_* value (14.72 kJ/mole) with chemisorption as its rate-limiting step. This study demonstrated the practical applicability of CCB as reactive material in the treatment of As(V)-contaminated groundwater.

## Figures and Tables

**Table 1:** Natural groundwater characteristics obtained from Taiwan.

Parameters	Unit	Values	Parameters	Unit	Values
pH		7.9	As(V)	µg/L	50.99
Conductivity	μS/cm	84.3	K^+^	mg/L	30.52
Eh	mV	131.2	Ca^2+^	mg/L	22.66
Dissolved oxygen	mg/L	3.1	Na^+^	mg/L	631.77
Resistivity	kΩ	11.2	Fe^2+^	mg/L	0.64
TOC	mg/L	4.3	Mg^2+^	mg/L	0.28
Turbidity	NTU	17.2	Cl^−^	mg/L	253.00
COD	mg/L	6.7	SO_4_^2−^	mg/L	36.34
TDS	mg/L	52.3	PO_4_^2−^	mg/L	67.17

**Table 2:** Values of coefficient of determination and kinetic parameters.

Kinetic Model	Parameter	Values
Pseudo-first order	*k*_1_ (min^−1^)	0.0117
	*q_e_*_,theo_ (µg/g)	2.201
	*R* ^2^	0.9013
	*χ* ^2^	31.26
	*RMSE*	22.05
Pseudo-second order	*k*_2_ (g/µg•min)	4.502 × 10^−3^
	*q_e_*_,theo_ (µg/g)	8.340
	*R* ^2^	0.9951
	*χ* ^2^	8.33
	*RMSE*	4.31
Intraparticle diffusion	*k_p_* (g/µg•min^1/2^)	1.519
	*C*	6.408
	*R* ^2^	0.8450
	*χ* ^2^	77.22
	*RMSE*	45.93
Film diffusion	*k_fd_* (min^−1^)	0.0353
	*R* ^2^	0.9256
	*χ* ^2^	77.22
	*RMSE*	45.93

**Table 3:** List of isotherm parameters for the removal of As(V) using CCB.

Isotherm	Parameters	Values
Langmuir	*K_L_* (L/mg)	0.0019
	*q_m_* (mg/g)	1.4660
	*R*_L_	11.6690
	*R* ^2^	0.9899
	*χ* ^2^	0.91
	*RMSE*	4.87
Freundlich	*n* (g/L)	0.2142
	*K_f_* (mg/g)	0.1538
	*R* ^2^	0.6508
	*χ* ^2^	11.03
	*RMSE*	18.75
D-R	*B* (mol^2^/kJ^2^)	0.6069
	*q_m_* (mg/g)	0.2704
	*E* (kJ/mole)	0.9077
	*R* ^2^	0.6259
	*χ* ^2^	13.83
	*RMSE*	22.64

**Table 4:** Comparison of adsorbents utilized in the removal of As(V).

Adsorbent	q_e_ (mg/g)	Reference
F400 (granular activated carbon)	1.01	Vitela-Rodriguez and Rangel-Mendez, 2013 [38]
Chitosan/glutaraldehyde	2.86	Gogoi et al., 2016 [30]
Clay/chitosan/glutaraldehyde	3.40	Gogoi et al., 2016 [30]
UltraCarb (activated carbon)	43.6	Chen et al., 2007 [36]
Polymer-clay nanocomposite ion exchange resin	55.0	Urbano et al., 2012 [39]
nZVI-zeolite	38.3	Suazo-Hernández et al., 2019 [37]
CCB	1.47	Present study

**Table 5:** Thermodynamic parameters for the removal of As(V) onto CCB.

Temperature (K)	*E_a_* (J/mol)	Δ*H*^0^ (J/mol)	Δ*S*^0^ (kJ/mol•K)	Δ*G*^0^ (kJ/mol)
298	14.72	8.31	29.10	3.89
308				3.32
318				3.31
328				2.99
